# Autoantibodies against the *N*-Methyl-d-Aspartate Receptor Subunit NR1: Untangling Apparent Inconsistencies for Clinical Practice

**DOI:** 10.3389/fimmu.2017.00181

**Published:** 2017-03-01

**Authors:** Hannelore Ehrenreich

**Affiliations:** ^1^Clinical Neuroscience, Max Planck Institute of Experimental Medicine, DFG Research Center for Nanoscale Microscopy and Molecular Physiology of the Brain (CNMPB), Göttingen, Germany

**Keywords:** blood–brain barrier dysfunction, immunoglobulin class, serum, cerebrospinal fluid, inflammation, functionality assays, neuropsychiatric diseases, healthy subjects

## Abstract

This viewpoint review provides an integrative picture of seemingly contradictory work published on *N*-methyl-d-aspartate receptor 1 (NMDAR1) autoantibodies (AB). Based on the present state of knowledge, it gives recommendations for the clinical decision process regarding immunosuppressive treatment. Brain antigen-directed AB in general and NMDAR1-AB in particular belong to a preexisting autoimmune repertoire of mammals including humans. Specific autoimmune reactive B cells may get repeatedly (perhaps transiently) boosted by various potential stimulants (e.g., microbiome, infections, or neoplasms) plus less efficiently suppressed over lifespan (gradual loss of tolerance), likely explaining the increasing seroprevalence upon aging (>20% NMDAR1-AB in 80-year-old humans). Pathophysiological significance emerges (I) when AB-specific plasma cells settle in the brain and produce large amounts of brain antigen-directed AB intrathecally and/or (II) in conditions of compromised blood–brain barrier (BBB), for instance, upon injury, infection, inflammation, or genetic predisposition (*APOE4* haplotype), which then allows substantial access of circulating AB to the brain. Regarding NMDAR1-AB, functional effects on neurons *in vitro* and elicitation of brain symptoms *in vivo* have been demonstrated for immunoglobulin (Ig) classes, IgM, IgA, and IgG. Under conditions of brain inflammation, intrathecal production and class switch to IgG may provoke high NMDAR1-AB (and other brain antigen-directed AB) levels in cerebrospinal fluid (CSF) and serum, causing the severe syndrome named “anti-NMDAR encephalitis,” which then requires immunosuppressive therapy on top of the causal encephalitis treatment (if available). However, negative CSF NMDAR1-AB results cannot exclude chronic effects of serum NMDAR1-AB on the central nervous system, since the brain acts as “immunoprecipitator,” particularly in situations of compromised BBB. In any case of suspected symptomatic consequences of circulating AB directed against brain antigens, leakiness of the BBB should be evaluated by CSF analysis (albumin quotient as proxy) and magnetic resonance imaging before considering immunosuppression.

This viewpoint review is arranged around two tabulated figures, one summarizing NMDAR1 autoantibody (AB) findings and integrating them into an explanatory model (Figure 1) and the other trying to give clear recommendations for the clinical decision process on immunosuppressive treatment based on the present state of knowledge (Figure 2).

**Figure 1:**
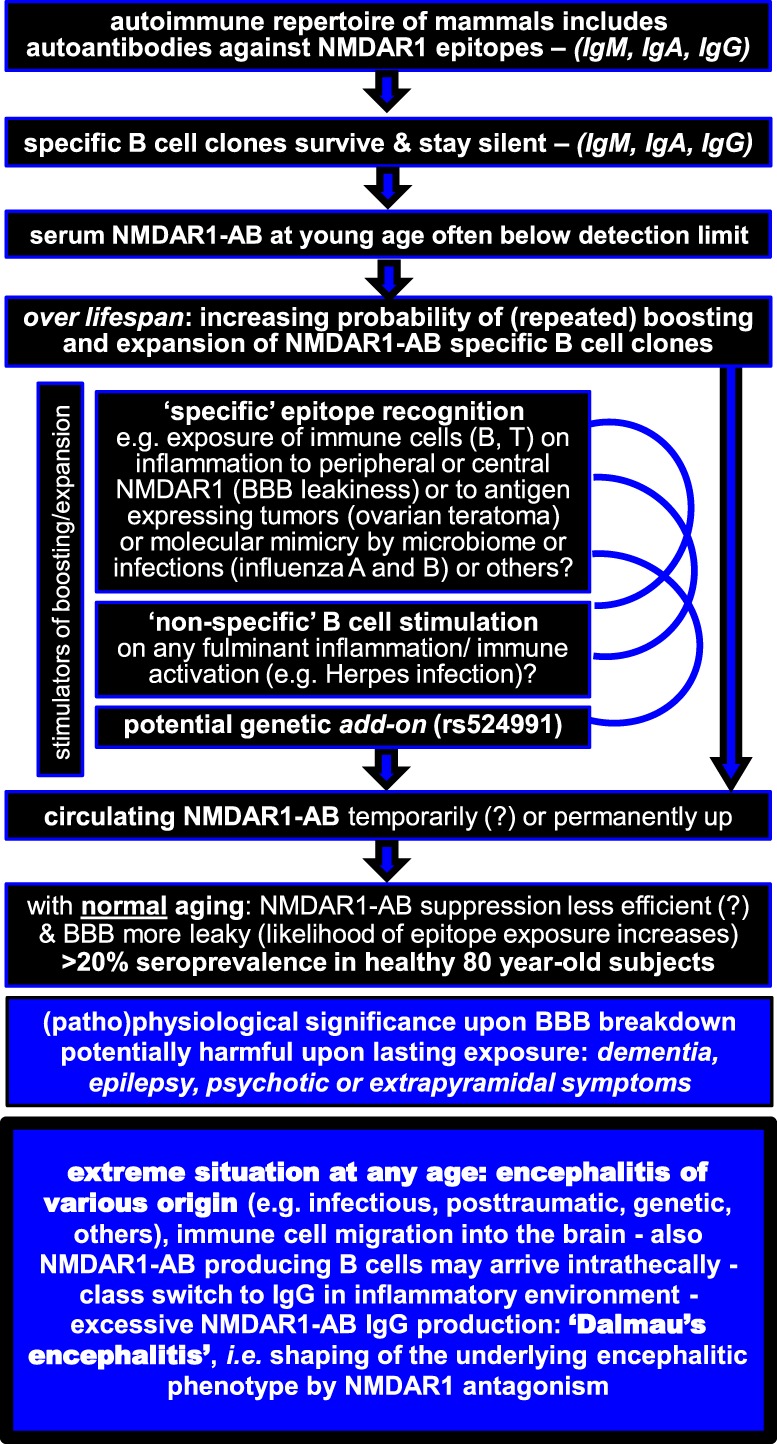
**Integration of NMDAR1 autoantibodies (AB) findings into an explanatory model**.

**Figure 2:**
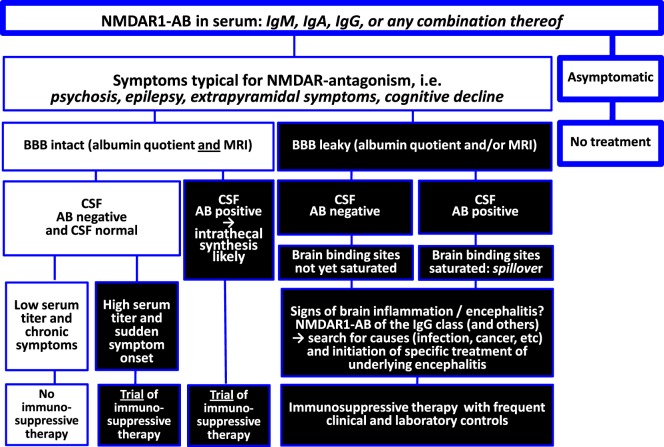
**Recommendations for the clinical decision process**.

Please note that the new nomenclature GluN1 for NMDAR1/NR1 is disregarded here for consistency with most of the respective reviewed literature.

## NMDA Receptors in Brain and Periphery

*N*-methyl-d-aspartate receptors (NMDAR) are glutamate-gated ion channels, abundantly expressed in mammalian brain (1). They form heteromers of NR1, NR2, and NR3 subunits, with NR1 being the only obligatory partner. NMDAR are pivotal for regulating neuronal/synapse function and are also expressed by non-neuronal cell types in the brain like astrocytes, oligodendrocytes, or endothelial cells (2–5). In addition, peripheral expression has been reported, e.g., in the gastrointestinal tract or in immune cells (6).

## Anti-NMDAR Encephalitis

Autoantibodies of the immunoglobulin G (IgG) class directed against NMDAR1 have been originally linked with a condition named “anti-NMDAR encephalitis” (7–10). In 2007, Dalmau and colleagues first described a paraneoplastic syndrome, based on 12 women with ovarian teratoma, carrying IgG AB against NMDAR NR1/2 subunits. The syndrome variably consisted of psychosis, cognitive decline, epileptic seizures, dyskinesia, decreased consciousness, and autonomic instability. The authors reported in many subsequent publications, based on increasing numbers of individuals with anti-NMDAR encephalitis, high serum and cerebrospinal fluid (CSF) titers of NMDAR1-AB of the IgG class in this condition as well as frequently favorable response to immunosuppressive therapy (7–10). As syndrome-pertinent pathophysiological mechanism, NMDAR1-AB induced decrease of NMDAR-mediated currents, due to enhanced receptor internalization, and thus reduced surface expression, has been suggested (11). However, over several years, healthy subjects were not even investigated in appreciable numbers for NMDAR1-AB seroprevalence. Nevertheless, the presence of NMDAR1-AB of the IgG class in serum (not only in CSF) was and still is claimed to be disease specific (7–10), causing some confusion in the literature and unfortunately also in clinical practice.

## Syndromes Reminiscent of NMDAR1 Antagonism

Since NMDAR hypofunction had been hypothesized to be a central mechanism in schizophrenia, due to induction of psychotic symptoms by antagonists (12, 13), the question arose several years ago whether a subpopulation of schizophrenic subjects may be previously overlooked anti-NMDAR encephalitis cases. So far, the literature—mostly based on small sample sizes and following the original “disease-specificity claim of NMDAR1-AB of the IgG class”—yielded discordant results (14–20). Analogously, other pathological conditions, likewise reminiscent of NMDAR antagonism, e.g., epilepsy or dementia, were investigated for the presence of NMDAR1-AB. A flood of publications appeared—many of them case reports—describing associations of NMDAR1-AB with a wide variety of syndromes. Finally, NMDAR1-AB of other immunoglobulin (Ig) classes (IgM and IgA) were also reported to be associated with disease conditions (17, 21–23). An interesting question that has remained totally open up to now is whether NMDAR1-AB can also lead to “peripheral phenotypes,” considering the expression of NMDAR in peripheral organs and tissues (6).

## Equal Distribution of Serum NMDAR1-AB Across Health and Disease

Unexpectedly, recent work of us and others on together >5,000 individuals challenged the “disease-specificity claim” of any NMDAR1-AB by demonstrating age dependent up to >20% NMDAR1-AB seroprevalence, including IgM, IgA, and IgG, in both healthy and ill subjects. Interestingly, NMDAR1-AB of the IgE class were searched for but never detected (24). Diseases investigated in these studies comprise neuropsychiatric conditions (schizophrenia, affective disorders, Parkinson’s disease, amyotrophic lateral sclerosis, Alzheimer’s disease, stroke, multiple sclerosis, and personality disorders) as well as general medical conditions, e.g., diabetes or hypertension (24–28). Also NMDAR1-AB titer range in serum and the distribution of Ig classes were comparable across all investigated disease groups as well as healthy individuals (24–28). Any 40-year-old person has a ~10% and any 80-year-old person has a ~20% chance of displaying NMDAR1-AB seropositivity (24).

## Functionality of NMDAR1-AB

This surprising discovery raised the question of whether these AB are all functional. Since biochip mosaics and a cell-based assay, the clinical standard procedure (HEK293T-cells transfected with NMDAR1 and secondary AB against human IgG, IgM, or IgA; Euroimmun, Lübeck, Germany), were used for all of these NMDAR1-AB determinations (see also below), additional assays had to be performed to further consolidate these unanticipated findings by proving AB functionality. These *in vitro* assays (all conducted with sera following ammonium sulfate precipitation of immunoglobulins and dialysis) revealed similar effects of NMDAR1-AB—independent of the Ig class—on receptor internalization in human IPSC-derived neurons as well as in primary mouse neurons. Likewise, NMDAR1-AB of all Ig classes reduced glutamate-evoked currents in NR1-1b/NR2A expressing *Xenopus laevis* oocytes (26, 28, 29). *In vivo* studies in mouse and human suggest comparable effects of serum NMDAR1-AB of all Ig classes regarding modulation of brain functions (see more details below).

## Methods of AB Detection—Still Room for Improvement

A still pending problem calling for standardization is the diversity of methods applied for AB determination with different specificity and sensitivity. Regarding NMDAR1-AB (where we have the most solid own experience), cell-based assays are certainly the superior method to detect NMDAR1-AB since epitopes are exposed in a natural way to enable AB to specifically detect them. But even these assays differ, with some authors using transiently transfected live cells accepting their potential variability and batch-to-batch variation problems, versus others using fixed and permeabilized cells expressing the whole NMDAR1 subunit, likely allowing better standardization (Euroimmun). This latter assay is currently being used throughout the world to diagnose NMDAR1-AB encephalitis. Based on our own experience with this assay in association with functionality studies performed in parallel (receptor internalization, electrophysiology, and *in vivo* studies), it appears to be the most reliable method at this point. It is, however, strongly recommended to use this assay in combination with secondary AB that are highly specific for the various Ig classes (anti-human IgG, anti-human IgA, and anti-human IgM) since cross-reacting AB may lead to wrong conclusions regarding, e.g., the prevalence of IgG AB. The use of rat, mouse, human, or monkey brain sections for immunohistochemical detection of specific AB may be a helpful addition providing supportive evidence. In contrast, the typical ELISA based on peptides cannot be recommended as a detection method for NMDAR1-AB, since many false-positive and/or false-negative results may be obtained due to the unnatural (removed from the position in the cell membrane) epitope exposure. These assays seem only suitable for follow-up analyses, for instance, the determination of the AB titer course using a series of samples from the same donor, previously clearly diagnosed as seropositive by cell-based and functional assays.

## A Decisive Role of the Blood–Brain Barrier (BBB) for Syndromic Relevance

Wondering why so many serum NMDAR1-AB carriers remain healthy, we hypothesized that a compromised BBB might decide on their pathophysiological significance. Importantly, enhanced BBB permeability may differ regionally, thereby explaining individually variable symptomatic consequences (30). As an animal model, we studied *ApoE^−/−^* mice with known BBB leakage in comparison to wild-type littermates (31). Intravenous injection of purified Ig fractions from NMDAR-AB seropositive (IgM, IgG, and IgA) human subjects led to alterations in spontaneous open field activity and hypersensitive (psychosis related) response to MK-801 in the open field exclusively in *ApoE^−/−^* mice (28). Exploring the role of a compromised BBB subsequently also in humans, we saw indeed more severe neurological symptoms in NMDAR1-AB carriers (of any Ig class) with a history of birth complications or neurotrauma, conditions with likely chronically leaky BBB (28). Along the same lines, we investigated *APOE4* carriers since the *APOE4* haplotype has been associated with a permeable BBB (32, 33). We obtained first hints that NMDAR-AB may enhance delusions of grandiosity and mania in neuropsychiatrically ill *APOE4* carriers, which are then more likely diagnosed schizoaffective (29). A modifier role of preexisting circulating NMDAR1-AB (again of all classes) was also seen in human ischemic stroke. In patients with intact BBB before occurrence of the insult, NMDAR1-AB were protective with respect to evolution of lesion size, whereas in *APOE4* carriers, NMDAR1-AB were associated with larger insult volumes (24). These findings emphasize that not only degree but also duration of BBB dysfunction, acute versus chronic, may play a pivotal role in syndrome shaping by NMDAR1-AB.

## The Brain as Immunoprecipitator of NMDAR1-AB

Circulating NMDAR1-AB of all Ig isotypes temporarily decreased after stroke (24). This led us hypothesize that brain tissue with its densely expressed NMDAR1 (accessible after BBB breakdown) may act as a trap for circulating NMDAR1-AB (25). We first addressed the question of whether serum NMDAR1-AB would be detectable in the CSF. Of *N* = 271 middle-aged subjects (diagnosed with multiple sclerosis or disease controls) with CSF–serum pairs available, 26 were NMDAR1-AB seropositive (which is in the expected range) but, remarkably, only 1 was CSF positive. In contrast, tetanus-AB (omnipresent due to obligatory vaccination but not binding to brain tissue) were present in serum and CSF of all subjects, with CSF levels higher upon compromised BBB. Translational experiments in mice proved the hypothesis that the brain acts as “immunoprecipitator”: simultaneous injection of NMDAR1-AB IgG and a non-brain-binding “non-sense-AB” (anti-GFP IgG) resulted in high detectability of the former only in the brain (distinctly more pronounced upon BBB dysfunction) and the latter only in CSF (25). These data may help explaining potential symptomatic consequences of serum AB directed against brain antigens. Whereas leakiness of the BBB has a major role and should be evaluated in cases where pathological relevance of circulating NMDAR1-AB is suspected, negative results regarding AB titers in CSF cannot automatically exclude brain effects.

## Epitopes Recognized by NMDAR1-AB

The next question was whether these apparently overall functional NMDAR1-AB would recognize the same epitope and whether this could potentially explain their high seroprevalence. Again unexpectedly, epitope mapping using seven different NMDAR1 constructs revealed recognition by NMDAR1-AB-positive sera of different epitopes, located in the extracellular ligand binding and the *N*-terminal domain (NTD) as well as the intracellular *C*-terminal and the extra large pore domain. NMDAR1-AB seropositivity was polyclonal/polyspecific in half of the investigated sera and likely mono or oligoclonal/oligospecific (mainly IgG) in the other half. Overall, no particular disease-related pattern appeared: NMDAR1 epitopes were comparable across disease groups (26). Published work on NMDAR1-AB epitopes has been scarce before this systematic investigation and had focused on IgG recognizing NTD and the NTD-G7 domain (N368/G369), probably because this region and Ig class were first deemed pathognomonic for anti-NMDAR encephalitis (8, 34). Indeed, it seems that factors predisposing young women [including those with ovarian teratoma and with lupus erythematosus (35)] to neuropsychiatric manifestations of NMDAR1-associated autoimmunity are connected with NTD or NTD-G7 epitopes. The accentuated role of IgG in this context is still a matter of speculation but likely related to inflammation-induced class switch in the brain (36).

## Predisposing Factors to Carry or Boost NMDAR1-AB

On the basis of these *in vitro* and *in vivo* findings, we have to assume that basically all naturally occurring NMDAR1-AB have pathogenic potential irrespective of epitope and Ig class. This does, however, not mean that the type of Ig class cannot initiate distinct cascades of secondary events and thereby further shape the ultimate tissue response. But now even more questions arise: How can we explain the disease-independent high seroprevalence of NMDAR1-AB, increasing with age? Do we know of any predisposing factors, and if so, how can we integrate their role into the full picture? NMDAR1-AB were initially associated with oncological conditions (teratoma) (7). Later on, a predisposition to carry these AB was seen upon influenza A and B seropositivity, a finding replicated in an independent sample (25, 28). Also a genome-wide significant genetic marker, rs524991, even related to NMDAR biology, was found associated with NMDAR1-AB (28). Whether a leaky BBB, causing enhanced exposure of central NMDAR1 to cells of the immune system, can induce NMDAR1-AB formation and/or boost preexisting specific B cell clones is presently unclear and needs to be systematically investigated. Another attractive idea that has not yet been pursued in the NMDAR1-AB field is the potential modulatory influence of the microbiome on boosting of NMDAR1-AB (37).

## Other Brain Antigen-Directed AB

Why do we see NMDAR1-AB so abundantly in health and disease? Does this also hold true for other AB directed against brain antigens? To address these questions, we analogously studied 24 other brain antigen-directed serum AB, previously connected with pathological conditions. Again to some surprise, this work revealed comparable frequency, titers, and Ig class distribution in healthy and ill subjects. Seroprevalence, however, of all of these 24 AB was distinctly lower (<2%) in contrast to NMDAR1-AB (up to >20%) (27). Strikingly, the predominant Ig class did not depend on health or disease state either, but on antigen location, with intracellular epitopes predisposing to IgG (27). The equal distribution of these 24 other AB in health and disease is less astonishing when considering that multiple brain-directed AB have been reported in serum of healthy humans and of different other mammalian species (38, 39) as well as abundantly in CSF of encephalitis cases (40), even though the respective brain antigens were not specified. To sum up, brain antigen-directed AB in general and NMDAR1-AB in particular seem to be part of a preexisting autoimmune repertoire (37, 41–44) that gains (patho)physiological significance in conditions of intrathecal synthesis or compromised BBB, for instance, upon injury, infection, brain inflammation, or genetic predisposition to BBB leakiness (*APOE4* haplotype).

## Conclusions and Recommendations

All naturally occurring serum NMDAR1-AB obviously have pathogenic potential. For still widely unexplored reasons, they are highly frequent (more than other so far identified brain-directed AB), and their prevalence clearly increases with age. NMDAR-AB seropositivity alone definitely does not justify immunosuppressive treatment. Syndromal relevance of serum NMDAR1-AB depends on accessibility to the brain, i.e., BBB permeability. Moreover, brain inflammation likely plays a crucial role in determining syndrome acuteness and severity as contributed by circulating NMDAR1-AB and even more pronounced by respective plasma cells that reside in or potentially migrate to the brain in inflammatory conditions to produce AB intrathecally (40). In the inflammatory milieu, they are boosted upon epitope exposure and experience class switch to IgG (36). Findings in individuals with herpes encephalitis may further support this view (22, 45).

Any underlying encephalitis, be it infectious, lesion induced, genetic, or “idiopathic”, may undergo prominent syndrome shaping by the presence of NMDAR1-AB in the sense of “Dalmau’s encephalitis” (7–10), which then requires immunosuppressive therapy on top of the causal encephalitis treatment (if available). Whether intrathecally produced NMDAR1-AB alone, without any underlying preexisting inflammation, can cause “Dalmau’s encephalitis” remains to be determined. In any case of otherwise suspected symptomatic consequences of serum AB directed against brain antigens in the absence of overt encephalitis, leakiness of the BBB should be evaluated. Since the albumin quotient (employed as clinical approximation to diagnose BBB breakdown) rather indicates blood–CSF barrier disturbance and may not always be pathological in mild cases of BBB leakiness (46–48), additional determination of BBB disruption (global or local) by a novel magnetic resonance imaging (MRI) method (47), which can be established as *add-on* to routine contrast-enhanced MRI, may prove helpful for estimating necessity and benefit especially of extended immunosuppressive therapeutic interventions.

## Author Contributions

The author confirms being the sole contributor of this work and approved it for publication.

## Conflict of Interest Statement

The author declares that the research was conducted in the absence of any commercial or financial relationships that could be construed as a potential conflict of interest.
